# Juvenile-Onset Recurrent Rhabdomyolysis Due to Compound Heterozygote Variants in the *ACADVL* Gene

**DOI:** 10.3390/brainsci13081178

**Published:** 2023-08-08

**Authors:** Beatrice Labella, Gaetana Lanzi, Stefano Cotti Piccinelli, Filomena Caria, Simona Damioli, Barbara Risi, Enrica Bertella, Loris Poli, Alessandro Padovani, Massimiliano Filosto

**Affiliations:** 1 Department of Clinical and Experimental Sciences, University of Brescia, 25100 Brescia, Italy; beatrice.labella93@gmail.com (B.L.); stefano.cottipiccinelli@centrocliniconemo.it (S.C.P.); alessandro.padovani@unibs.it (A.P.); 2 Unit of Neurology, ASST “Spedali Civili”, 25100 Brescia, Italy; loris.poli@asst-spedalicivili.it; 3 Medical Genetics Laboratory, Diagnostic Department, ASST-Pedali Civili of Brescia, 25100 Brescia, Italy; gaetana.lanzi@asst-spedalicivili.it; 4 NeMO—Brescia Clinical Center for Neuromuscular Diseases, 25064 Brescia, Italy; filomena.caria@centrocliniconemo.it (F.C.); simona.damioli@centrocliniconemo.it (S.D.); barbara.risi@centrocliniconemo.it (B.R.); enrica.bertella@centrocliniconemo.it (E.B.)

**Keywords:** lipid myopathy, *ACADVL*, VLCAD, VLCADD, myoglobinuria, rhabdomyolysis

## Abstract

Very long-chain acyl-CoA dehydrogenase (VLCAD) deficiency is a rare autosomal recessive long-chain fatty acid oxidation disorder caused by mutations in the *ACADVL* gene. The myopathic form presents with exercise intolerance, exercise-related rhabdomyolysis, and muscle pain, usually starting during adolescence or adulthood. We report on a 17-year-old boy who has presented with exercise-induced muscle pain and fatigue since childhood. In recent clinical history, episodes of exercise-related severe hyperCKemia and myoglobinuria were reported. Electromyography was normal, and a muscle biopsy showed only “moth-eaten” fibers, and a mild increase in lipid storage in muscle fibers. NGS analysis displayed the already known heterozygote c.1769G>A variant and the unreported heterozygote c.523G>C change in *ACADVL* both having disease-causing predictions. Plasma acylcarnitine profiles revealed high long-chain acylcarnitine species levels, especially C14:1. Clinical, histopathological, biochemical, and genetic tests supported the diagnosis of VLCAD deficiency. Our report of a novel pathogenic missense variant in *ACADVL* expands the allelic heterogeneity of the disease. Since dietary treatment is the only therapy available for treating VLCAD deficiency and it is more useful the earlier it is started, prompt diagnosis is essential in order to minimize muscle damage and slow the disease progression.

## 1. Introduction

The *ACADVL* gene, located on chromosome 17p.13.1, encodes very long-chain acyl-CoA de-hydrogenase (VLCAD) [1]. This protein targets the inner mitochondrial membrane, where it catalyzes the initial step of the fatty acid beta-oxidation pathway, specifically the catalysis of 14- to 20-carbon acyl-CoAs in mitochondria [1].

VLCAD deficiency (VLCADD; OMIM #201475) is a very rare clinically heterogeneous disorder in which high plasma levels of long-chain acylcarnitine conjugates, especially the tetradecenoyl (C14:1) acylcarnitine, are found [1]. In the past, the estimated incidence was reported to range from 1 in 30,000 to 1 in 100,000 worldwide [1]. However, it seems to be much higher thanks to novel screening tools such as newborn screening (NBS), which can detect even milder forms that were previously difficult to diagnose [2,3,4]. According to the variable expression of the disease, three major phenotypes have been described [1,5]: infantile onset presents at birth or within the first months of life with hypertrophic cardiomyopathy, recurrent episodes of hypoketotic hypoglycemia, dicarboxylic aciduria, and hepatic failure which leads to a high mortality rate (50–75%) [1,5]; childhood onset occurs from late infancy to early childhood (around 4 years old), manifesting with recurrent episodes of hypoketotic hypoglycemia, dicarboxylic aciduria, and minimal or any cardiac involvement [1,4]; the later-onset phenotype is characterized by exercise-induced myoglobinuria, which may begin in childhood or early adulthood [1,5]. 

Missense mutations or in-frame deletions are usually associated with milder forms of VLCADD, while *ACADVL* null alleles are found in severe neonatal onset [5,6]. 

NBS based on blood spot acylcarnitine levels (typically C14:1 acylcarnitine) has given new information about the genotype–phenotype relationship and disclosed several novel pathogenetic variants [4,7]. 

VLCADD may benefit from a specific low-fat/high-carbohydrate diet, avoiding fasting and triheptanoin; a nutrition management guideline has already been published [8,9,10]. Since dietary treatment is more useful the earlier it is started, it is important to achieve an early diagnosis, which is difficult to obtain, especially in late-onset forms. 

In this study, we report the case of an adolescent presenting with recurrent rhabdomyolysis episodes and harboring two heterozygous variants in the *ACADVL* gene, and we emphasize the importance of thinking about this disease in the differential diagnostic process to quickly reach a correct diagnosis and start the dietary treatment as soon as possible.

## 2. Case Presentation

The patient was a 17-year-old boy who was the third child of non-consanguineous Moroccan parents.

Familial history was characterized by maternal familiarity for hereditary cerebral cavernous angiomas.

The patient had an uncomplicated antenatal period and was born by caesarean section.

The neonatal examination was unremarkable. Growth and psychomotor development were regular.

At 5 years old, the patient started complaining of recurrent abdominal pain. As lactose intolerance was suspected, a dairy-free diet was introduced with no significant benefits.

At 13 years old, the patient was hospitalized for an acute onset of headache, vomiting, and intense myalgias in the lower limbs after a basketball game. He was diagnosed with acute myositis with rhabdomyolysis. In the following year, the patient suffered several mild episodes of inappetence and vomiting.

At 15 years old, after drinking alcohol, he experienced drowsiness, headache and emesis, painful abdomen, and nausea. Biochemistry laboratory tests showed elevation of CK levels (12394 U/L) and serum creatinine (0.91 mg/dL). Toxicological exams were negative except for ethanolemia (0.9 g/dL). Abdomen FAST-ultrasound was performed with no signs of hepato-splenomegalia. Hypertransaminasemia (aspartate transaminase 734 U/L, alanine transaminase 170 U/L) and increased levels of lactate dehydrogenase (LDH 751 U/L) were also observed. Immunological and infectious tests resulted negative. Blood morphology showed no abnormalities, excluding hemolytic anemia. 

Electromyography of the four limbs did not show pathological patterns.

**Figure 1:**
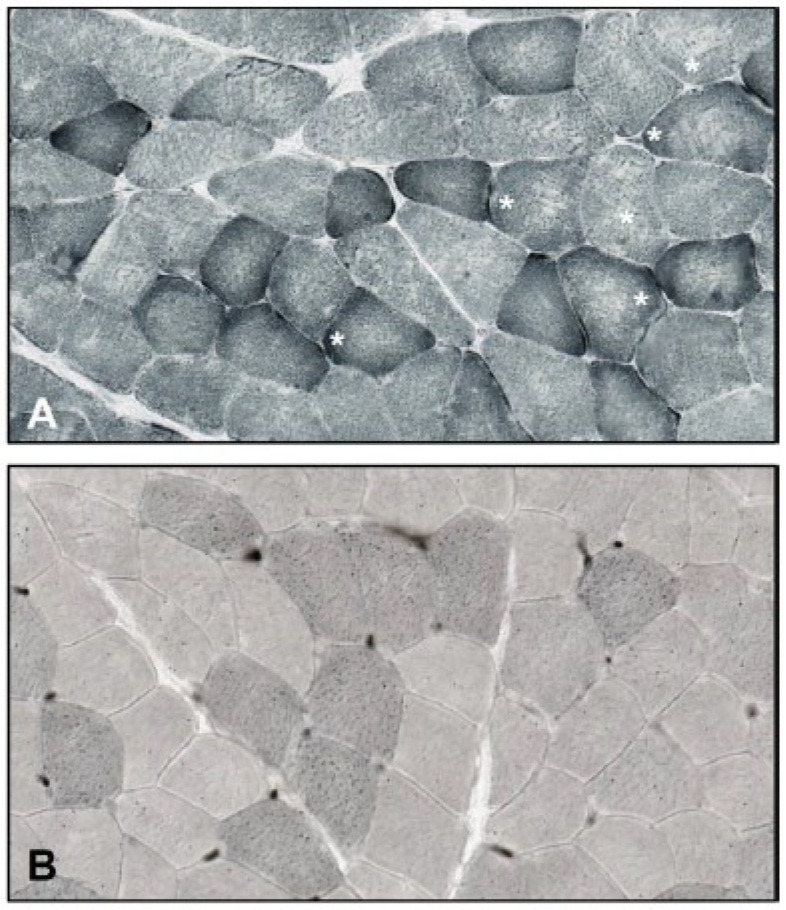
(**A**) Skeletal muscle biopsy revealing some “moth-eaten” fibers at NADH staining (asterisks). (**B**) Sudan Black staining showing only mild lipid droplets in scattered muscle fibers.

Muscle biopsy was performed on suspicion of metabolic myopathy. It showed a predominance of type 2 fibers, a few moth-eaten fibers at the oxidative stains (Figure 1A) and a minimal increase in lipid droplets in a few fibers from Sudan Black staining (Figure 1B). PAS staining was normal.

**Figure 2:**
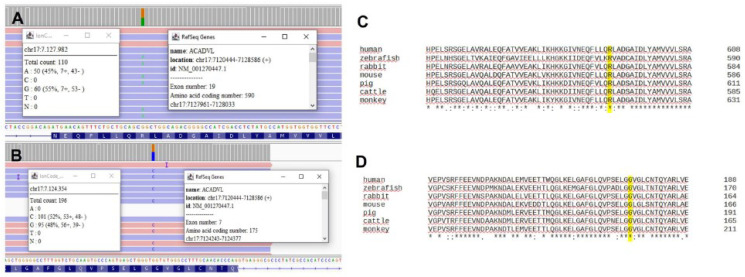
NGS results for the two heterozygote variants in the *ACADVL* gene and phylogenetic analysis for the related amino acid changes. (**A**) c.1769G>A; (**B**) c.523G>C; (**C**) phylogenetic analysis showed highly conserved residue Arg at the 590 site; (**D**) phylogenetic analysis showed highly conserved residue Gly at the 175 site.

Next generation sequencing (NGS) by a 34-gene panel for metabolic myopathies revealed the heterozygote c.523G>C variant and the heterozygote c.1769G>A change in *ACADVL* (NM_001270447.1) both having disease-causing prediction and resulting in the amino acid changes p.Gly175Arg and Arg590Gln, respectively (Figure 2). 

The healthy mother and father were heterozygous for c.523G>C and c.1769G>A, respectively. 

Plasma acylcarnitine profile revealed high long-chain acylcarnitine species levels, especially C14:1.

A nutritional-based therapeutic approach was used in accordance with management recommendations for VLCADD [8]. Nutritional intervention aimed to minimize the production of abnormal fatty acid metabolites (limiting the intake of long-chain fatty acids, LCFAs) and provided a source of energy (medium-chain triglycerides, MCTs) which can bypass the enzymatic block [8]. 

Clear information about the dietary regimen was provided and the importance of avoiding going without food for extended periods of time was stressed.

## 3. Discussion

The *ACADVL* gene is about 8142 bp long (GRCh38.p14). It contains 23 exons, which encode for a protein with a 40-amino acid leader peptide, yielding a mature 615-residue protein [11,12]. Alternative splicing results in multiple transcript variants encoding different isoforms [11,12]. 

VLCAD differs from other acyl-CoAs dehydrogenases because it acts as a dimer and contains an additional stretch of 180 amino acids at its C-terminus that is comprised composed of an -helical bundle [13]. Glu-422 functions as the key catalytic residue [5]. A structurally dynamic subdomain of the VLCAD C-terminus is required for direct membrane interaction [13]. 

Around 180 pathogenic and likely pathogenic variants have been reported in ClinVar database (https://www.ncbi.nlm.nih.gov/clinvar, accessed on 24 June 2023) while about 60 have been reported in the Global Variome shared LOVD database (https://databases.lovd.nl/shared/genes/ACADVL, accessed on 24 June 2023). 

One of the variants identified in our patient, c.1769G>A, has been reported in ClinVar with interpretations ranging from uncertain significance (VUS) (one submitter) to pathogenic (five submitters). Mutation Taster classifies this variant as disease causing. The amino acid change p.Arg590Gln changes a wild-type positively R-charged amino acid with a polar amino acid in the C-terminal region, affecting protein folding and stability [14]. 

The second identified variant, c.523G>C, has not been reported in variant databases. It results in the amino acid change p.Gly175Arg leading to an arginine (positively R-charged) to glycine (non-polar) substitution. It is predicted to affect not only the helix domain but also the catalytic region. 

Our description is useful for some reasons:

First, it expands the allelic heterogeneity of the disease by confirming the pathogenicity of the c.1769G>A substitution and by reporting a novel missense variant in *ACADVL* that can be classified as pathogenic. Moreover, we report a juvenile-onset VLCADD which is a rare and scarcely known condition. For this reason, it could be underdiagnosed, even though it should always be taken into account in the differential diagnosis of rhabdomyolysis in young adults. 

Rhabdomyolysis is a life-threatening condition characterized by severe acute muscle injury resulting in muscle pain, weakness, and/or swelling with release of myofiber contents into the bloodstream, which develop over hours to days [15]. Serum CK levels exceeding five times the upper limit of normal are the cut-off usually accepted to define rhabdomyolysis [15]. Acute renal failure (ARF) is the most important complication, mostly related to moderate–severe rhabdomyolysis [15]. 

Diagnostic work-up of rhabdomyolysis should always rule out systemic or acquired etiologies such as muscle trauma, metabolic disturbances, endocrine disorders (e.g., hyperthyroidism), or medications [16,17]. Once all these causes are properly excluded, the patient’s history and their family history, along with a neurological examination, should guide the following diagnostic steps [16,17].

Exercise history should be investigated in cases of episodic or recurrent rhabdomyolysis [16]. A detailed description of the timing of the onset of symptoms during exercise is fundamental if metabolic myopathy is suspected [16]. Glycogen storage disorders should be suspected in cases of onset symptoms after short bursts of high-intensity exercise or isometric activity [17,18]. Symptoms onset after prolonged submaximal activity or fasting are suggestive of fatty acid oxidation disorders as a reflection of the metabolic switch of energy production from glycolysis to fatty acid metabolism [17,18]. 

Clinical manifestations of VLCADD range from severe systemic involvement to mild episodic symptoms, thus leading to frequent misdiagnosis or “non-diagnosis” [19]. Especially, the adult-onset phenotype may be clinically non-specific, and this results in a frequent diagnostic delay [19]. The patient is usually asymptomatic, and serum CK may be normal between acute attacks, thus inducing non-expert practitioners to rule out a muscular disease [15]. Even when a muscular disease is suspected, a muscle biopsy may show a very mild or absent accumulation of lipid, thus making a pathological diagnosis difficult [18]. 

The expanded usage of NBS based on dried blood spot acylcarnitine levels has raised awareness of VLCADD, and it is an efficient screening tool not only for early diagnosis of affected subjects but also for detecting unaffected carriers [7,20]. However, it represents only a recent screening tool, so it is important for clinicians to detect juvenile/adult patients that had no opportunity to be screened at birth or come from countries where NBS has not yet been introduced.

The most important issue is to think of VLCADD as a possible cause of rhabdomyolysis in adults. A correct exercise and timing onset analysis as well as the fasting-related onset of episodes may help in suspecting a fatty acid oxidation defect, and then a combination of confirmatory tests including the acylcarnitine profile and gene sequencing may achieve the correct diagnosis [18].

Based on age, severity, and clinical history, a specific dietary fat composition should be defined as part of the therapeutic approach. In this regard, evidence- and consensus-based guidelines for nutrition management have been developed recently [8].

If a dietary regimen is correctly introduced, no specific limitations in physical exercise are necessary so that VLCADD patients with a milder phenotype can be physically active with some precautions (e.g., hydration, consuming MCTs before exercise) [8].

Supplementation with L-carnitine is still debated because of the potential cardiac risk of accumulation of long-chain acylcarnitines, so it should be avoided in acute illness and in chronic management unless the free carnitine concentration is <10 μmol/L [8].

The potential benefit of higher protein intake in VLCADD is under investigation, as a few studies suggest it may improve body composition and metabolic stability [8,21].

Avoidance of fasting, having meals and snacks spaced throughout the day, is a general recommendation, and a maximum interval of 10 to 12 h of fasting is set after the first year of life [8].

Triheptanoin, an oral liquid triglyceride with three medium-odd-chain heptanoic acids, has been studied as a substitute for MCTs in the treatment of VLCADD, showing improvement in cardiac function, muscle strength, exercise tolerance, and/or reduction in metabolic decompensation [8,22,23]. In June 2020, it was officially approved in the USA as a treatment for long-chain fatty acid disorders [24]. Triheptanoin should be administered at mealtimes or snacks with a target daily dosage of up to 35% of the patient’s total prescribed daily calorie intake divided into 4 doses or more [24]. The most common adverse effects are gastrointestinal (vomiting, diarrhea, and abdominal pain) [25]. A Phase 3 randomized, double-blind trial on young VLCADD patients (up to 17 years old) is currently recruiting and aims to determine the effect of triheptanoin compared with MCT on major clinical events.

Bezafibrates are peroxisome proliferator-activated receptor (PPAR) pan-agonists that have shown potential improvement of fatty acid oxidation capacities in deficient VLCAD-fibroblasts by enhancing the residual level of mutant enzyme activity via gene expression stimulation [26,27]. However, results from clinical trials have been controversial, and further studies are required to assess their effectiveness in VLCADD [28,29,30].

Coenzyme Q10, an essential cofactor in oxidative phosphorylation in mitochondria and cell membranes, supports continuous oxidation–reduction cycles and acts as a major lipid-soluble antioxidant and anti-inflammatory agent through the regulation of gene expression [31]. In the past, its administration has shown improvement in the perception of fatigue in patients experiencing disease-related fatigue (e.g., fibromyalgia) and in healthy subjects [31]. Riboflavin, or vitamin B2, plays a role in mitochondrial energy metabolism and stress responses along with its derivates (flavin mononucleotide and flavin adenine dinucleotide) [32]. Administration of coenzyme Q10 and riboflavin in VLCADD is now limited to case reports in conjunction with other treatment modalities, but no randomized studies have proved their effectiveness [8,33,34].

## 4. Conclusions

In conclusion, clinicians should “think metabolic” in case of recurrent rhabdomyolysis in adolescence or young adults after exclusion of systemic or acquired etiologies. Since late-onset presentation is exceedingly rare, VLCADD is often not considered, and the patient is not referred to a neuromuscular disease center. Prompt diagnosis is essential for starting a specific diet early and avoiding prolonged fasting and strenuous exercise in order to minimize muscle damage and slow the disease progression.

## Data Availability

Data are available upon justified request.

## References

[1] Leslie N.D., Saenz-Ayala S., Adam M.P., Mirzaa G.M., Pagon R.A., Wallace S.E., Bean L.J.H., Gripp K.W., Amemiya A. (2009). Very Long-Chain Acyl-Coenzyme A Dehydrogenase Deficiency. GeneReviews^®^.

[2] Boneh A., Andresen B.S., Gregersen N., Ibrahim M., Tzanakos N., Peters H., Yaplito-Lee J., Pitt J.J. (2006). VLCAD deficiency: Pitfalls in newborn screening and confirmation of diagnosis by mutation analysis. Mol. Genet. Metab..

[3] Bleeker J.C., Kok I.L., Ferdinandusse S., Van der Pol W.L., Cuppen I., Bosch A.M., Langeveld M., Derks T.G.J., Williams M., De Vries M. (2019). Impact of newborn screening for very-long-chain acyl-CoA dehydrogenase deficiency on genetic, enzymatic, and clinical outcomes. J. Inherit. Metab. Dis..

[4] Remec Z.I., Groselj U., Drole Torkar A., Zerjav Tansek M., Cuk V., Perko D., Ulaga B., Lipovec N., Debeljak M., Kovac J. (2021). Very Long-Chain Acyl-CoA Dehydrogenase Deficiency: High Incidence of Detected Patients with Expanded Newborn Screening Program. Front. Genet..

[5] Andresen B.S., Olpin S., Poorthuis B.J., Scholte H.R., Vianey-Saban C., Wanders R., Ijlst L., Morris A., Pourfarzam M., Bartlett K. (1999). Clear correlation of genotype with disease phenotype in very-long-chain acyl-CoA dehydrogenase deficiency. Am. J. Hum. Genet..

[6] Chen T., Tong F., Wu X.Y., Zhu L., Yi Q.Z., Zheng J., Yang R.L., Zhao Z.Y., Cang X.H., Shu Q. (2020). Novel ACADVL variants resulting in mitochondrial defects in long-chain acyl-CoA dehydrogenase deficiency. J. Zhejiang Univ. Sci. B.

[7] Miller M.J., Burrage L.C., Gibson J.B., Strenk M.E., Lose E.J., Bick D.P., Elsea S.H., Sutton V.R., Sun Q., Graham B.H. (2015). Recurrent ACADVL molecular findings in individuals with a positive newborn screen for very long chain acyl-coA dehydrogenase (VLCAD) deficiency in the United States. Mol. Genet. Metab..

[8] Van Calcar S.C., Sowa M., Rohr F., Beazer J., Setlock T., Weihe T.U., Pendyal S. (2020). Nutrition management guideline for very-long chain acyl-CoA dehydrogenase deficiency (VLCAD): An evidence- and consensus-based approach. Mol. Genet. Metab..

[9] Yamada K. (2019). Management and diagnosis of mitochondrial fatty acid oxidation disorders: Focus on very-long-chain acyl-CoA dehydrogenase deficiency. J. Hum. Genet..

[10] Shiraishi H., Yamada K., Egawa K. (2021). Efficacy of bezafibrate for preventing myopathic attacks in patients with very long-chain acyl-CoA dehydrogenase deficiency. Brain Dev..

[11] Zhou C., Blumberg B. (2003). Overlapping gene structure of human VLCAD and DLG4. Gene.

[12] Aoyama T., Souri M., Ueno I., Kamijo T., Yamaguchi S., Rhead W.J., Tanaka K., Hashimoto T. (1995). Cloning of human Very-Long-Chain Acyl-Coenzyme A Dehydrogenase and molecular characterization of its deficiency in two patients. Am. J. Hum. Genet..

[13] Prew M.S., Camara C.M., Botzanowski T., Moroco J.A., Bloch N.B., Levy H.R., Seo H., Dhe-Paganon S., Bird G.H., Herce H.D. (2022). Structural basis for defective membrane targeting of mutant enzyme in human VLCAD deficiency. Nat. Commun..

[14] Schiff M., Mohsen A.W., Karunanidhi A., McCracken E., Yeasted R., Vockley J. (2013). Molecular and cellular pathology of very-long-chain acyl-CoA dehydrogenase deficiency. Mol. Genet. Metab..

[15] Chavez L.O., Leon M., Einav S., Varon J. (2016). Beyond muscle destruction: A systematic review of rhabdomyolysis for clinical practice. Crit. Care.

[16] Harmelink M. (2022). Uncommon Causes of Rhabdomyolysis. Crit. Care Clin..

[17] Nance J.R., Mammen A.L. (2015). Diagnostic evaluation of rhabdomyolysis. Muscle Nerve.

[18] Lilleker J.B., Keh Y.S., Roncaroli F., Sharma R., Roberts M. (2018). Metabolic myopathies: A practical approach. Pract. Neurol..

[19] Fatehi F., Okhovat A.A., Nilipour Y., Mroczek M., Straub V., Töpf A., Palibrk A., Peric S., Rakocevic Stojanovic V., Najmabadi H. (2020). Adult-onset very-long-chain acyl-CoA dehydrogenase deficiency (VLCADD). Eur. J. Neurol..

[20] Loeber J.G., Platis D., Zetterström R.H., Almashanu S., Boemer F., Bonham J.R., Borde P., Brincat I., Cheillan D., Dekkers E. (2021). Neonatal Screening in Europe Revisited: An ISNS Perspective on the Current State and Developments Since 2010. Int. J. Neonatal. Screen..

[21] Evans M., Andresen B.S., Nation J., Boneh A. (2016). VLCAD deficiency: Follow-up and outcome of patients diagnosed through newborn screening in Victoria. Mol. Genet. Metab..

[22] Roe C.R., Brunengraber H. (2015). Anaplerotic treatment of long-chain fat oxidation disorders with triheptanoin: Review of 15 years experience. Mol. Genet. Metab..

[23] Tucci S., Floegel U., Beermann F., Behringer S., Spiekerkoetter U. (2017). Triheptanoin: Long-term effects in the very long-chain acyl-CoA dehydrogenase-deficient mouse. J. Lipid. Res..

[24] Shirley M. (2020). Triheptanoin: First Approval. Drugs.

[25] Vockley J., Burton B., Berry G., Longo N., Phillips J., Sanchez-Valle A., Chapman K., Tanpaiboon P., Grunewald S., Murphy E. (2021). Effects of triheptanoin (UX007) in patients with long-chain fatty acid oxidation disorders: Results from an open-label, long-term extension study. J. Inherit. Metab. Dis..

[26] Djouadi F., Aubey F., Schlemmer D., Ruiter J.P., Wanders R.J., Strauss A.W., Bastin J. (2005). Bezafibrate increases very-long-chain acyl-CoA dehydrogenase protein and mRNA expression in deficient fibroblasts and is a potential therapy for fatty acid oxidation disorders. Hum. Mol. Genet..

[27] Gobin-Limballe S., Djouadi F., Aubey F., Olpin S., Andresen B.S., Yamaguchi S., Mandel H., Fukao T., Ruiter J.P., Wanders R.J. (2007). Genetic basis for correction of very-long-chain acyl-coenzyme A dehydrogenase deficiency by bezafibrate in patient fibroblasts: Toward a genotype-based therapy. Am. J. Hum. Genet..

[28] Ørngreen M.C., Vissing J., Laforét P. (2015). No effect of bezafibrate in patients with CPTII and VLCAD deficiencies. J. Inherit. Metab. Dis..

[29] Ørngreen M.C., Madsen K.L., Preisler N., Andersen G., Vissing J., Laforêt P. (2014). Bezafibrate in skeletal muscle fatty acid oxidation disorders: A randomized clinical trial. Neurology.

[30] Shiraishi H., Yamada K., Oki E., Ishige M., Fukao T., Hamada Y., Sakai N., Ochi F., Watanabe A., Kawakami S. (2019). Open-label clinical trial of bezafibrate treatment in patients with fatty acid oxidation disorders in Japan; 2nd report QOL survey. Mol. Genet. Metab. Rep..

[31] Testai L., Martelli A., Flori L., Cicero A.F.G., Colletti A. (2021). Coenzyme Q10: Clinical Applications beyond Cardiovascular Diseases. Nutrients.

[32] Mosegaard S., Dipace G., Bross P., Carlsen J., Gregersen N., Olsen R.K.J. (2020). Riboflavin Deficiency-Implications for General Human Health and Inborn Errors of Metabolism. Int. J. Mol. Sci..

[33] Laforêt P., Acquaviva-Bourdain C., Rigal O., Brivet M., Penisson-Besnier I., Chabrol B., Chaigne D., Boespflug-Tanguy O., Laroche C., Bedat-Millet A.L. (2009). Diagnostic assessment and long-term follow-up of 13 patients with Very Long-Chain Acyl-Coenzyme A dehydrogenase (VLCAD) deficiency. Neuromuscul. Disord..

[34] Scalais E., Bottu J., Wanders R.J., Ferdinandusse S., Waterham H.R., De Meirleir L. (2015). Familial very long chain acyl-CoA dehydrogenase deficiency as a cause of neonatal sudden infant death: Improved survival by prompt diagnosis. Am. J. Med. Genet. A.

